# Comparative Chloroplast Genomes of *Zosteraceae* Species Provide Adaptive Evolution Insights Into Seagrass

**DOI:** 10.3389/fpls.2021.741152

**Published:** 2021-09-23

**Authors:** Jun Chen, Yu Zang, Shuai Shang, Shuo Liang, Meiling Zhu, Ying Wang, Xuexi Tang

**Affiliations:** ^1^College of Marine Life Sciences, Ocean University of China, Qingdao, China; ^2^Laboratory for Marine Ecology and Environmental Science, Qingdao National Laboratory for Marine Science and Technology, Qingdao, China; ^3^Key Laboratory of Marine Eco-Environmental Science and Technology, First Institute of Oceanography, Ministry of Natural Resources, Qingdao, China; ^4^College of Biological and Environmental Engineering, Binzhou University, Binzhou, China

**Keywords:** *Zosteraceae*, seagrass, chloroplast genome, genome structure, adaptive evolution

## Abstract

Seagrasses are marine flowering plants found in tropical and sub-tropical areas that live in coastal regions between the sea and land. All seagrass species evolved from terrestrial monocotyledons, providing the opportunity to study plant adaptation to sea environments. Here, we sequenced the chloroplast genomes (cpGenomes) of three *Zostera* species, then analyzed and compared their cpGenome structures and sequence variations. We also performed a phylogenetic analysis using published seagrass chloroplasts and calculated the selection pressure of 17 species within seagrasses and nine terrestrial monocotyledons, as well as estimated the number of shared genes of eight seagrasses. The cpGenomes of *Zosteraceae* species ranged in size from 143,877 bp (*Zostera marina*) to 152,726 bp (*Phyllospadix iwatensis*), which were conserved and displayed similar structures and gene orders. Additionally, we found 17 variable hotspot regions as candidate DNA barcodes for *Zosteraceae* species, which will be helpful for studying the phylogenetic relationships and interspecies differences between seagrass species. Interestingly, nine genes had positive selection sites, including two ATP subunit genes (*atpA* and *atpF*), two ribosome subunit genes (*rps4* and *rpl20*), two DNA-dependent RNA polymerase genes (*rpoC1* and *rpoC2*), as well as *accD*, *clpP*, and *ycf2*. These gene regions may have played key roles in the seagrass adaptation to diverse environments. The Branch model analysis showed that seagrasses had a higher rate of evolution than terrestrial monocotyledons, suggesting that seagrasses experienced greater environmental pressure. Moreover, a branch-site model identified positively selected sites (PSSs) in *ccsA*, suggesting their involvement in the adaptation to sea environments. These findings are valuable for further investigations on *Zosteraceae* cpGenomes and will serve as an excellent resource for future studies on seagrass adaptation to sea environments.

## Introduction

Seagrasses are marine flowering plants in class Monocotyledoneae that form vast meadows, and flower and seed underwater. As opposed to lower plants such as macroalgae (seaweeds), seagrasses are higher angiosperms that can live fully in seawater. They have terrestrial plant origins and reentered the sea millions of years ago (Orth et al., 2006). The globally recognized seagrasses have been divided into 6 families and 13 genera (∼74 species), while China mainly contains 4 families and 10 genera (22 species), accounting for ∼30% of the global seagrasses (Short et al., 2011; Huang et al., 2016). Seagrass beds are formed by large-scale seagrasses, which may consist of either a single or multiple species, and possess important ecological functions, such as stabilizing coastal sediments and thereby maintaining water quality, providing diverse habitats for several unique organisms, serving as nurseries for marine organisms (fish, crustaceans, and mollusks), and providing nutrients (N and P) and organic carbon to other parts of the oceans, including the deep sea, as well as contribute significantly to carbon sequestration (Suchanek et al., 1985; Hemminga and Duarte, 2000; Duarte et al., 2005; Orth et al., 2006).

The general trend of biological evolution is from low to high (from aquatic to terrestrial), but some unique evolutionary events occur during the evolutionary process, such as “second entry into water.” Some species have gradually changed from terrestrial to aquatic after their ancestors separated from their terrestrial relatives (sea otters, cetaceans, pinnipeds, and seagrasses). The ancestors of seagrasses separated from terrestrial monocotyledons 70–100 million years ago and have since adapted to submerged marine life (Waycott et al., 2007). During this process, a series of adaptive changes took place in their morphological structures and physiological ecology (Ye, 2002). As members of a polyphyletic group of plants, seagrasses live in the tidal and subtidal regions of marine habitats (Kato et al., 2003). Seagrasses are mainly found in the transition zone between the sea and terrestrial environments, which experiences some of the greatest environmental pressure on Earth. Owing to the long-term dual and interactive effects of the sea and terrestrial environments, there is ample opportunity to explore and uncover complex and diverse adaptive mechanisms and evolutionary information. However, to date, seagrass studies have mainly focused on species investigations, distribution monitoring, and ecological restoration (Short and Coles, 2001; Orth et al., 2006; Short et al., 2011). With the development of sequencing technology, hotspots in the field of evolutionary biology have aimed to reveal the evolutionary adaptive mechanisms of seagrasses at the molecular or genomic level. Olsen et al., conducted the first genomic study in 2016 on *Zostera marina* and found that *Z. marina* has lost genes for regulating stomatal opening, terpenoid and ethylene synthesis, ultraviolet and infrared sensing, and plant pigment that are common in terrestrial angiosperms for adapting to the marine environment. Additionally, *Z. marina* has acquired unique genes that regulate ion metabolism, nutrient absorption, gas exchange, and other life activities, which have allowed for the adaptation to the complex marine environment (high salt, high osmotic pressure, and low light intensity) (Olsen et al., 2016; Williams, 2016). Moreover, in 2016, the complete genome of *Z. muelleri* was sequenced and it was found that, to adapt to the marine environment, genes related to hormone biosynthesis, signal transduction, and cell wall catabolism were either lost or modified (Lee et al., 2016).

Chloroplasts are multifunctional organelles in plant cells that play critical roles in photosynthesis and carbon fixation (Wicke et al., 2011; Daniell et al., 2016). The cpGenomes have a typical quadripartite structure with a pair of inverted repeats (IRs) separated by a small single-copy (SSC) and a large single-copy (LSC) region (Sugiura, 1992). Most angiosperm cpGenomes are remarkably conserved in terms of structure, gene content, and order (Wicke et al., 2011). Generally, plant cpGenomes are recombination-free, maternally inherited, and have low rates of nucleotide substitutions, which make them valuable sources of genetic markers for phylogenetic and population genetic analyses (Korpelainen, 2004; Ravi et al., 2008). Adaptive evolution is defined as the improved adaptability of a species to changing environmental conditions during the evolutionary process. Given the conservation of the cpGenome, studies on these genomes will enhance our understanding of plant adaptive evolution. To our knowledge, a comparative analysis on the cpGenomes of seagrasses that have adapted to the sea environment has not yet been performed.

In this study, we aimed to provide comprehensive insights into the evolution of the cpGenomes of several seagrass species. First, we sequenced the cpGenomes of three *Zosteraceae* species and conducted comparative cpGenome analyses using these three genomes and previously annotated cpGenomes of *Z. marina*. Then, we constructed a phylogenetic tree using the cpGenomes of all published seagrass genomes obtained from the NCBI database. Finally, we estimated positive selection for seagrass species and detected the type of selection pressure (positive, neutral, and purifying) to address whether they experienced different evolutionary forces compared to terrestrial monocotyledons.

## Materials and Methods

### Sample Collection and Sequencing Assembly

Samples of three seagrass species (*Z. nigricaulis*, *Phyllospadix iwatensis*, and *Z. japonica*) were collected from their natural habitat with *Z. japonica* (120.6801 E, 37.93384 N) and *Phyllospadix iwatensis* (120.6476 E, 37.97221 N) collected from Yantai, China, and *Z. nigricaulis* (144.415392 E, 38.142656 S) collected from Geelong, Australia, respectively. All samples are currently stored at the Marine Ecology Laboratory of Ocean University of China. Total DNA was extracted from fresh leaves using TRIzol^®^ Reagent (Invitrogen). Library construction for Illumina sequencing was performed using an Illumina TruSeq^TM^ Nano DNA Sample Prep Kit. Total DNA was sequenced using an Illumina HiSeq 4000 platform (150 bp^∗^2) (Shanghai BIOZERON Co., Ltd., Shanghai, China). Then, we removed potential low-quality reads from the raw reads using Trimmomatic 0.39 software (Bolger et al., 2014). NOVOPlasty v2.7.2 software (Dierckxsens et al., 2017) was used to assemble the cpGenomes using *Z. marina* as the reference sequence. GapCloser software (Luo et al., 2012) was used to fill the remaining local inner gaps and correct the single base polymorphisms for the final assembly results. Finally, four junctions between the IRs and SSC/LSC regions were confirmed using the reference sequence.

### Chloroplast Genome Annotation

The cpGenomes were annotated using GeSeq (Tillich et al., 2017) and annotation results were checked with BLAST and DOGMA (Wyman et al., 2004). Then, all protein-coding sequences were BlastP against the non-redundant (Nr) NCBI, SwissProt, KEGG, and COG databases for their functional annotations. Then, a circular map of the three seagrass species was obtained using Organellar Genome DRAW (Greiner et al., 2019). Finally, the newly obtained chloroplast genomes of three *Zosteraceae* species were submitted into the GenBank under accession numbers were MZ576842, MZ573775, MZ571509, respectively.

### Comparative Chloroplast Genome Analysis

The cpGenome structures of the four *Zosteraceae* species (including *Z. marina*) were compared using mVISTA software (Frazer et al., 2004). Subsequently, we extracted all coding regions and intergenic spacers (IGSs) to examine the regions of divergence within the four *Zosteraceae* species for further phylogenetic analysis. The percentage of variable sites was calculated within each homologous region. Then, the expansion and contraction of the IR regions were analyzed by comparing the positions of the LSC/IR and SSC/IR junctions, and their adjacent genes using IRscope (Amiryousefi et al., 2018).

### Phylogenetic Relationships

Phylogenetic analysis of eight seagrass cpGenomes from order Alismatales was performed using *Oryza minuta* as the outgroup. All shared plastid coding genes (PCGs) were concatenated into a super matrix and aligned using MUSCLE (Edgar, 2004). The evolutionary history was estimated using the maximum likelihood (ML) method based on the JTT + G + I + F model with 1,000 bootstrap replicates using MEGA X software (Kumar et al., 2018).

### Adaptive Evolution Analysis

We compared the rate of non-synonymous (*d*_*N*_) and synonymous (*d*_*S*_) substitutions using the CODEML program, implemented by PAML v4.7 (Yang, 2007), to quantify the selective pressure. Ratios of *dN/dS* were calculated, where ω = 1, ω > 1, and ω < 1 indicated neutral, positive, and purifying selection, respectively. We used the species phylogenetic tree obtained by previous studies (Davis et al., 2013; Petersen et al., 2016; Ross et al., 2016; Givnish et al., 2018) as the input tree file (Supplementary Figure 1).

To detect positively selected sites (PSSs) in eight seagrass species, we used two site-specific models (M7 and M8) in the CODEML program. A likelihood ratio test (LRT) with χ^2^ distribution was performed to determine and compare nested models with a *p* < 0.05 significance threshold. Bayes empirical Bayes (BEB) analysis was used to identify sites under positive selection with posterior probabilities > 0.80 (Ziheng et al., 2005). PSSs were identified using the Datamonkey web server (Pond and Frost, 2005) based on three methods, the FEL, SLAC, and MEME models, with a *p* < 0.1 significance threshold. In this study, positive selection sites detected by ≥ 3 methods were considered positive selection sites.

To determine whether each shared PCG faced a different evolutionary force in different habitats, Branch and branch-site models were conducted to detect positive selection in the eight seagrasses species, which were collectively set as the foreground branch. The LRT with χ^2^ distribution was performed to determine and compare the nested models with a *p* < 0.05 significance threshold. BEB analysis was used to identify sites under positive selection with posterior probabilities > 0.80.

## Results

### Genomic Features of *Zosteraceae* Species

**FIGURE 1:**
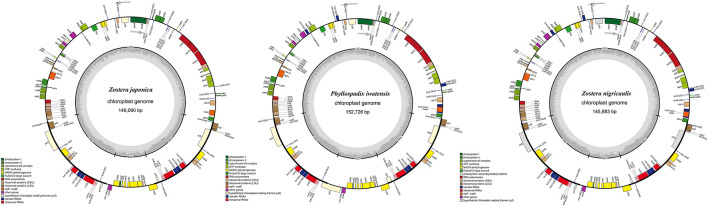
Gene maps of three *Zosteraceae* species chloroplast genomes. Genes belonging to different functional groups are color-coded, as indicated.

The cpGenome sizes of the three species and *Z. marina* of family *Zosteraceae* ranged from 143,877 bp in *Z. marina* to 152,726 bp in *P. iwatensis*, which were composed of four regions, including an LSC and SSC region separated by two IRs (Figure 1 and Table 1). The length differences of the genomes were mainly determined by the SSC length. The GC content of the cpGenome sequences ranged from 35.46 to 36.18% and the highest GC content was 36.18% in *P. iwatensis*, followed by *Z. japonica* (35.89%), *Z. nigricaulis* (35.88%), and *Z. marina* (35.46%). The number of genes present in the 4 plastomes of the *Zosteraceae* species was similar, except for *Z. marina*, which lost some genes and thus had a reduced gene number. There were 132 genes in *P. iwatensis*, 131 in *Z. nigricaulis*, 127 in *Z. japonica*, and 116 in *Z. marina*, which had the lowest number of genes. *Z. nigricaulis* and *P. iwatensis* had 38 tRNA genes, while *Z. japonica* and *Z. marina* had 34 and 30, respectively. All species had eight rRNA genes. The number of PCGs ranged from 78 to 86. Among all genes, there were 7–19 single intron genes, and *ycf3*, *clpP* and two *rps12* (except for *Z. marina*) had two introns each.

**TABLE 1:** Chloroplast genome features of four *Zosteraceae* species.

Species name	Genome size (bp)	LSC size (bp)	IR size (bp)	SSC size (bp)	GC (%)	Total no. of genes	PCGs No	tRNA No	rRNA No	Single intron	Double intron
*Zostera nigricaulis*	145,883	83,541	24,430	13,482	35.88	131	85	38	8	19	4
*Phyllospadix iwatensis*	152,726	84,753	25,167	17,639	36.18	132	86	38	8	19	4
*Zostera japonica*	146,090	83,664	24,628	13,169	35.89	127	85	34	8	13	4
*Zostera marina*	143,877	83,224	25,915	8,823	35.46	116	78	30	8	7	2

### Boundary Regions and Comparative Analysis

**FIGURE 2:**
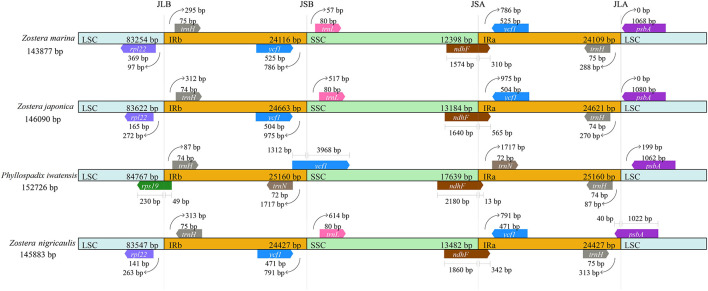
Comparison of the border positions of LSC, SSC, and IR regions in the chloroplast genomes in four *Zosteraceae* species.

There were 4 boundaries between the two IRs, and LSC and SSC in the cpGenome, namely, IRa-SSC (JSA), IRa-LSC (JLA), IRb-LSC (JLB), and IRb-SSC (JSB). When comparing the cpGenomes of the *Zosteraceae* species (Figure 2), we found that the four boundaries of the cpGenomes were relatively conserved. The JLB border was located between *rpl22* and *trnH*, except in *P. iwatensis*, which cut through *rps19*, resulting in a pseudogene. The JSB border was between *ycf1* and *trnL* in the 3 *Zostera* species, where *ycf1* was within the IRb. *P. iwatensis* had a 1,312-bp pseudogene. The JSA border was within *ndhF*, resulting in a 13–565-bp pseudogene in four species. Moreover, *psbA* was located entirely in the LSC and across the JLA border in *Z. japonica*, resulting in a 40-bp pseudogene in the IRa.

**FIGURE 3:**
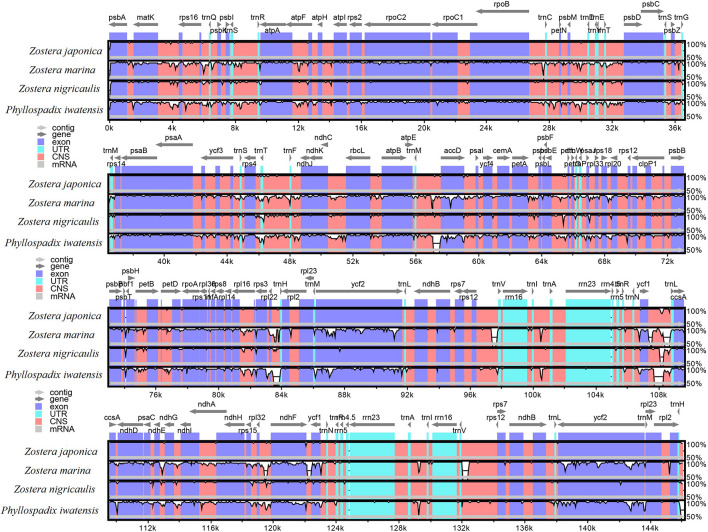
Sequence identity plots based on four *Zosteraceae* species.

**FIGURE 4:**
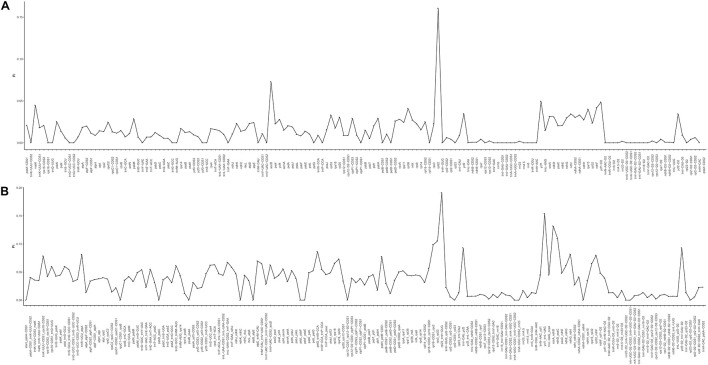
Percentages of variable characters in homologous regions among the chloroplast genomes of four *Zosteraceae* species. **(A)** Coding region. **(B)** The introns and spacers (IGS). The homologous regions are oriented according to their locations in the chloroplast genome.

The mVISTA program was used to analyze the overall sequence identity of the cpGenome of the *Zosteraceae* species, using the *Z. japonica* annotation as a reference (Figure 3). The cpGenomes were conserved and displayed similar structures and gene orders. The divergence level of the non-coding regions was higher than the coding regions. The IGS regions had the highest levels of divergence. Additionally, the LSC and SSC regions had a larger divergence than the IR regions (Figure 4).

### Phylogenetic Relationships

**FIGURE 5:**
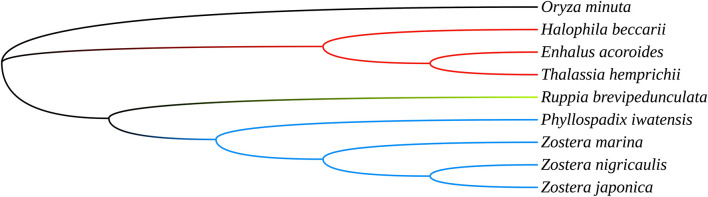
The ML tree of seagrasses based on all shared plastid coding genes.

To investigate the phylogenetic relationship of the seagrasses, we constructed an ML tree using MEGA X. As can be seen from the tree (Figure 5), the shared PCGs divided into three major clades: *Hydrocharitaceae*, *Zosteraceae*, and *Ruppiaceae*, among which, *Zosteraceae* and *Ruppiaceae* had a relatively close relationship. Moreover, *Z. marina* was a sister species to *Z. nigricaulis* and *Z. japonica* within *Zosteraceae*. These results showed that the phylogenetic proximity of all shared PCGs was closely related to the traditional taxonomic group (Ross et al., 2016).

### Adaptive Evolution Analysis

Model M8 and the SLAC, FEL, and MEME methods were used to detect specific codons under selection in 59 single-copy shared genes of 8 seagrass species (Supplementary Table 1). Sites identified by ≥ 3 methods were regarded as robust candidates for positive selection sites. A total of nine genes with at least one common positively selected codon were identified, including 2, 2, and 13 common positively selected codons in *accD*, *clpP*, and *ycf2*, respectively.

The results of the Branch model analysis of all shared PCGs showed that the ω values (ω = 0.16933) were significant (*p* < 0.01), indicating that at the whole-chloroplast protein level, monocotyledonous plants were strongly purified and selected for during their evolution (Table 2). Moreover, the ω values were significantly higher in seagrass groups than in terrestrial monocotyledonous groups (Table 2), indicating that the chloroplast protein-encoding genes in seagrasses evolved more rapidly than in terrestrial monocotyledons. Furthermore, the analysis of 54 single-copy shared genes showed that 23 genes had significantly different evolutionary rates between the two groups and all evolved rapidly in seagrass groups, except *cemA* and *psbT* (Supplementary Table 2).

**TABLE 2:** Selection pressure test results of all shared protein-coding genes in monocotyledons through a Branch model analysis.

Model	np	*LnL*	ω for branch	Model compared	*P*-values
A: All the branch has same ω	34	−176110.3	ω = 0.16933		
B: All the branch has same ω = 1	33	−184904.03	ω = 1	B vs. A	*p* < < 0.01
C: Clade of Seagrass has a ω_1_; other clade has a ω_2_	35	−175986.49	ω_1_ = 0.21950 ω_2_ = 0.14204	C vs. A	*p* < < 0.01

A total of 54 single-copy genes were also used for selective pressure estimation in the branch-site model analysis (Supplementary Table 3). Only Model A and Model A Null of *ccsA* were significant (*p* = 0.022). The BEB posterior probability of an amino acid site (213K) was > 0.80.

## Discussion

### Chloroplast Genome Features

In this study, the newly sequenced three *Zosteraceae* cpGenomes and previously published cpGenomes of *Z. marina* were roughly similar in size, gene order, and composition, and ranged in size from 143,877 to 152,726 Kb. The cpGenomes of the 4 species were comparable in size to other monocotyledons (Huotari and Korpelainen, 2012) and their sequences were highly conserved with no gene rearrangement events detected. Previous studies showed that gene loss was a common phenomenon among parasitic, submerged, and carnivorous plant plastomes (Wakasugi et al., 1994; Braukmann et al., 2009; Wicke et al., 2011), and was usually associated with functional transfers to the nucleus (Fleischmann et al., 2011). Similar events were also detected in our study, where *rps19* was completely lost in *Zostera*, and Hydrocharitaceae lost several NDH-related genes (Supplementary Table 4). Plastid NDH-related genes code for most of the subunits of the plastid NADH dehydrogenase enzyme complex that play a role in photooxidative stress responses (Martín and Sabater, 2010). Interestingly, previous studies have also shown a significant loss of NDH-related genes in the family of Hydrocharitaceae (Ross et al., 2016). Therefore, we speculate that this gene loss may be due to the adaptation to specific seagrass habitats and some genes may have been transferred to the nucleus. Previous studies showed that GC content is an important indicator of species affinity and GC skewness is an indicator of DNA lagging chains, leading chains, replication origin, and replication termini (Tillier and Collins, 2000; Necşulea and Lobry, 2007). Generally, in this study, plastomes were characterized by a low GC content in species from order Alismatales and the lowest GC content was detected in *Zosteraceae*. Moreover, the overall GC content of the IR region in *Zosteraceae* was higher than the LSC and SSC regions, a phenomenon that is common in other plants (Fan et al., 2018; Guo et al., 2018). Thus, we hypothesized that this may be caused by the presence of four RNA genes (*rrn16*, *rrn23*, *rrn4.5*, and *rrn5*) in the IR region.

### Sequence Variations

During cpGenome evolution, the expansion and contraction of IR boundaries allow for some genes to enter IR regions, while other genes can enter single-copy sequences, but the degree of sequence replication at the boundaries of each species is different (Yan et al., 2017; Fan et al., 2018; Wu et al., 2020). Our study showed that, although the cpGenomes of family *Zosteraceae* had a conserved structure, there were significant size differences caused mainly by IR boundary shifts (Figures 2, 3). For example, *ycf1* in *P. iwatensis* spanned the SSC/IRB region and spanned 3,968 bp in the SSC region, but was greatly reduced and located in the IR region of the other three species. Moreover, gene loss in *Zosteraceae* also caused IR boundary shifts. A previous study showed that the LSC/IRb border lies within the *rps19* and t*rnH-GUG* gene cluster in many monocot plastomes (Wang et al., 2008). However, in our study, due to the loss of *rps19*, the boundary of three *Zosteraceae* species, except *P. iwatensis*, was located between *rpl22* and *trnH-GUG*. To determine the divergence hotspots, we compared the whole cpGenome sequences of the *Zosteraceae* species to compute the percentages of variable characters in coding and non-coding regions (Figure 4). Our results indicated that the proportion of variable sites was higher in the non-coding regions than the coding regions, which is in accordance with the results found for other taxa (Fan et al., 2018; Sun et al., 2020). Among the four regions of the cpGenome, the sequence variation in the IR region was the lowest, while the variation in the LSC region was the highest. Thus, we speculated that the highly conserved IR region may be related to the higher GC content. Considering the proportion and number of variable sites, we propose 17 (*rpl22*, *accD*, *rpl22-trnH-GUG*, *ycf1-trnL-UAG*, *ccsA-ndhD*, *ndhD-psaC*, *rps3-rpl22*, *rpl16-rps3*, *trnI-CAU-ycf2*, *ycf2-trnI-CAU*, *petG-trnW-CCA*, *ndhG-ndhI*, *trnR-UCU-atpA*, *rpl32-ndhF*, *trnK-UUU_rps16*, *psbH-petB*, and *rps18-rpl20*) of the most variable hotspot regions as candidate DNA barcodes for *Zosteraceae*, which will aid future studies on the phylogenetic relationships and interspecies differences of seagrass species.

### Adaptive Selection

In our study, we found clear signatures of positive selection sites in nine genes shared by the eight seagrass species, which were identified as robust candidate sites using three methods. These genes included two ATP subunit genes (*atpA* and *atpF*), two ribosomal subunit genes (*rps4* and *rpl20*), two DNA-dependent RNA polymerase genes (*rpoC1* and *rpoC2*), as well as *accD*, *clpP*, and *ycf2* (Supplementary Table 1). ATP synthase is essential for plant photosynthesis and is usually a product of two genetic systems in plants (Westhoff et al., 1985). Six ATP subunit genes (*atpA*, *atpB*, *atpE*, *atpF*, *atpH*, and *atpI*) are encoded and synthesized in chloroplasts, and two genes exhibited site-specific selection in this study. Additionally, 16 genes were identified that encode ribosomal subunits, of which, two were under positive selection. Moreover, *rpoC* mainly encodes the β subunit of RNA polymerase. In this study, *rpoC1* and *rpoC2* were under positive selection, which may lead to alterations in cell wall metabolism, possibly as a result of altered transcription (Bisson et al., 2012; Cai et al., 2021). ACCase catalyzes malonyl-CoA formation from acetyl-CoA and bicarbonate in the first committed step of *de novo* fatty acid synthesis (Rawsthorne, 2002). The plastid, ACCase, is composed of four gene products, which were thought to be coordinately expressed, and *accD* is only encoded in the plastid genome (β-CT), while others are nuclear (Ke et al., 2000; Lee et al., 2004). In this study, we identified PSSs in *accD*, which may have played key roles in seagrass fatty acid biosynthesis. Additionally, *clpP* plays an important role in plant cells. The main function of its product is polypeptide degradation and it is a member of a gene family within the cpGenome that encodes clpP proteases (Clarke, 1999; Kuroda and Maliga, 2003). A previous study showed that *clpP* was under positive selection in Dipsacales species (Fan et al., 2018). We also found that *ycf2* had 13 sites under positive selection. This gene is the largest chloroplast gene reported in angiosperms and has become a useful gene for assessing sequence variations and evolutionary processes in plants (Drescher et al., 2000; Huang et al., 2010). Positive selection of *ycf2* was also found to be involved in the adaptation of other species (Fan et al., 2018; Zhong et al., 2019; Wu et al., 2020). However, owing to its unknown function, *ycf2* is an excellent candidate gene for future studies on the adaptive evolution of seagrass species. Moreover, these positively selected genes may have played key roles in seagrass adaptation to various environments.

Previous studies have shown that the cpGenome of seed plants has a lower evolutionary rate than nuclear genes (Drouin et al., 2008). In this study, the Branch model results showed that monocotyledonous plants experienced strong purifying selection, indicating that the cpGenomes of monocotyledonous plants were relatively conserved during evolution (Table 2). Furthermore, the Branch model analysis of the shared PCGs were significantly different (*p* < 0.01) between Model A and Model C (Table 2). These results suggest that the foreground and background branches have different evolutionary rates, indicating that seagrasses had a higher rate of evolution than terrestrial monocotyledons. Seagrasses mainly grow in the transition zone between the marine and terrestrial environments. Due to long-term dual and mutual effects of the marine and terrestrial environments, their habitats undergo great energy fluctuations. With the change of the tides, they are periodically directly exposed to the air and susceptible to enhanced UV-B radiation. Previous studies have shown that environmental energy stimulates metabolism on many levels and it is known that energy-rich habitats are often characterized by higher evolutionary rates (Davies et al., 2004; Clarke and Gaston, 2006). Solar radiation, especially UV radiation, plays a direct mutagenic role and may accelerate molecular evolution (Rothschild, 1999; Willis et al., 2009). Thus, we speculated that the evolutionary differences between seagrasses and terrestrial monocotyledons may be because seagrasses faced greater selective pressure in their habitats. The analysis of single-copy shared genes also showed that most significantly different (21/23) genes evolved rapidly in seagrass groups, except *cemA* and *psbT* (Supplementary Table 2). Adaptive evolution is likely to occur in specific lineages. We conducted a branch-site model analysis to further detect whether selection was limited in seagrasses. We identified 1 positively selected site (213K) in *ccsA* (Supplementary Table 3), which mainly encodes a protein required for heme attachment to c-type cytochromes (Xie and Merchant, 1996), which may have allowed seagrasses to adapt to sea environments.

## Conclusion

In this study, we sequenced the cpGenomes of 3 seagrass species, which revealed the cpGenome features between *Zosteraceae* (*Z. nigricaulis*, *P. iwatensis*, *Z. japonica*, and *Z. marina*). We also uncovered the phylogenetic relationship and evolution of single-copy shared genes alongside published seagrass sequences from the NCBI database. Our analyses revealed that all *Zosteraceae* species shared similar genome structures, gene orders, and compositions, and significant size differences were caused mainly by IR boundary shifts and gene loss. Site-specific selection analysis showed that some of the coding sites of nine chloroplast genes (*atpA*, *atpF*, *rps4*, *rpl20*, *rpoC1*, *rpoC2*, *accD*, *clpP*, and *ycf2*) underwent protein sequence evolution. Additionally, different levels of selection detected between seagrass and terrestrial monocotyledons lineages suggest their involvement in adaptation to sea environments. Phylogenetic results showed that *Z. marina* is a sister species to *Z. nigricaulis* and *Z. japonica* within *Zosteraceae*, which is supported by the classification of this genus. These findings are valuable for further investigations on *Zosteraceae* cpGenomes and will serve as an excellent resource for future studies on seagrass adaptation to sea environments.

## Data Availability Statement

The chloroplast genomes of three *Zosteraceae* species data for this study were submitted into the GenBank under accession numbers were MZ576842, MZ573775, and MZ571509, respectively.

## Author Contributions

JC conceived, designed the study, and wrote the manuscript. JC and YZ performed the experiments. SS, SL, and MZ contributed materials and analysis tools. XT and YW revised the manuscript. All authors approved the final manuscript.

## Conflict of Interest

The authors declare that the research was conducted in the absence of any commercial or financial relationships that could be construed as a potential conflict of interest.

## Publisher’s Note

All claims expressed in this article are solely those of the authors and do not necessarily represent those of their affiliated organizations, or those of the publisher, the editors and the reviewers. Any product that may be evaluated in this article, or claim that may be made by its manufacturer, is not guaranteed or endorsed by the publisher.

## Supplementary Material

Supplementary Figure 1 Phylogenetic trees of 17 monocotyledonous species.

Supplementary Table 1 Positively selected sites of 59 single-copy genes shared by eight seagrass species.

Supplementary Table 2 Selection pressure test results of shared protein-coding genes in seagrasses through a Branch model analysis.

Supplementary Table 3 Selection pressure test results of shared protein-coding genes in seagrasses through a branch-site model analysis.

Supplementary Table 4 Protein-coding gene losses in eight seagrass species.
