# Erratum: Comparative Chloroplast Genomes of *Zosteraceae* Species Provide Adaptive Evolution Insights Into Seagrass

**DOI:** 10.3389/fpls.2021.811304

**Published:** 2021-11-26

**Authors:** Frontiers Production Office

**Affiliations:** Frontiers Media SA, Lausanne, Switzerland

**Keywords:** *Zosteraceae*, seagrass, chloroplast genome, genome structure, adaptive evolution

Owing to a production error, several authors were linked to the wrong affiliations in the article as originally published, as follows:

Jun Chen^1†^, Yu Zang^2†^, Shuai Shang^1,3^, Shuo Liang^1^, Meiling Zhu^1^, Ying Wang^1,4*^ and Xuexi Tang^1,4*^

^1^ College of Marine Life Sciences, Ocean University of China, Qingdao, China

^2^ College of Biological and Environmental Engineering, Binzhou University, Binzhou, China

^3^ Laboratory for Marine Ecology and Environmental Science, Qingdao National Laboratory for Marine Science and Technology, Qingdao, China

^4^ Key Laboratory of Marine Eco-Environmental Science and Technology, First Institute of Oceanography, Ministry of Natural Resources, Qingdao, China

The correct presentation of the authors and their affiliations is as follows:

Jun Chen^1†^, Yu Zang^3†^, Shuai Shang^1,4^, Shuo Liang^1^, Meiling Zhu^1^, Ying Wang^1,2*^, Xuexi Tang^1,2*^

^1^ College of Marine Life Sciences, Ocean University of China, Qingdao, China

^2^ Laboratory for Marine Ecology and Environmental Science, Qingdao National Laboratory for Marine Science and Technology, Qingdao, China

^3^ Key Laboratory of Marine Eco-Environmental Science and Technology, First Institute of Oceanography, Ministry of Natural Resources, Qingdao, China

^4^ College of Biological and Environmental Engineering, Binzhou University, Binzhou, China

The publisher apologizes for this mistake. The original article has been updated.

