# Supplementary material for: Comparative Chloroplast Genomes of Zosteraceae Species Provide Adaptive Evolution Insights Into Seagrass
Source: Front Plant Sci. 2021 Sep 23;12:741152. doi: 10.3389/fpls.2021.741152 (PMC8495015; doi:10.3389/fpls.2021.741152)
Supplement: Supplementary Figure 1 — Phylogenetic trees of 17 monocotyledonous species. [file Data_Sheet_1.zip › Supplementary Table S1.DOCX]

**Table S1** Positively selected sites of 59 single-copy genes shared by eight seagrass species.

| Gene | *Ln L* M7 | *Ln L* M8 | *P-value* | M8 | SLAC | FEL | MEME |
| --- | --- | --- | --- | --- | --- | --- | --- |
| *psbA* | -2046.99 | -2046.96 | 0.970 | NA | NA | NA | NA |
| *matK* | -4814.91 | -4814.54 | 0.691 | NA | NA | 55 273 486 | 75 155 366 369 472 486 |
| *psbK* | -426.35 | -425.66 | 0.501 | 17 | NA | NA | NA |
| *psbI* | -217.11 | -217.11 | 1 | NA | NA | NA | NA |
| *psbD* | -2122.36 | -2121.83 | 0.589 | ***4*** | NA | ***4*** | ***4*** 11 |
| *psbC* | -2708.28 | -2708.32 | 0.961 | 415 | NA | NA | NA |
| *rpoB* | -8323.74 | -8318.87 | 0.00767** | 36 237 350 361 805 | NA | 433 572 766 | 407 433 688 751 766 |
| *rpoC1* | -5605.35 | -5594.97 | 3.10E-05** | 140 142 144 163 165 276 296 ***306*** 568 | NA | ***306*** | 55 110 142 201 226 ***306*** 360 556 568 569 586 596 |
| *rpoC2* | -11395.09 | -11391.89 | 0.0407* | 299 528 661 666 742 749 ***964*** 1251 1288 1322 | NA | 666 834 903 ***964*** 975 1028 | 12 216 749 809 903 ***964*** 971 975 977 1011 1169 1266 1297 |
| *rps2* | -1855.62 | -1855.47 | 0.861 | NA | NA | 113 133 | 60 113 146 159 |
| *atpI* | -1554.63 | -1553.83 | 0.449 | 32 63 67 146 210 | NA | 67 210 | NA |
| *atpH* | -460.73 | -460.73 | 1 | NA | NA | NA | NA |
| *atpA* | -3860.17 | -3856.47 | 0.0247* | 61 253 308 ***383*** 424 484 | NA | ***383*** 424 | 5 ***383*** 424 459 |
| *atpF* | -1507.49 | -1501.14 | 0.00174** | ***49*** 50 52 55 | ***49*** | NA | 31 ***49*** 52 94 105 112 116 |
| *psbZ* | -341.27 | -341.27 | 1 | NA | NA | NA | NA |
| *rps14* | -768.51 | -768.01 | 0.606 | 11 36 37 38 | NA | 36 | 86 |
| *psaB* | -4370.12 | -4370.01 | 0.896 | NA | NA | 150 | 282 413 612 |
| *psaA* | -4557.59 | -4557.37 | 0.802 | 25 | NA | NA | NA |
| *ycf3* | -980.30 | -980.30 | 1 | NA | NA | NA | NA |
| *rps4* | -1558.81 | -1554.55 | 0.0141* | 32 43 149 155 ***158*** 181 182 183 187 | NA | ***158*** | 42 43 ***158*** |
| *atpE* | -1017.56 | -1016.63 | 0.394 | NA | NA | NA | 52 79 112 |
| *atpB* | -3269.35 | -3268.16 | 0.304 | 103 201 354 | NA | NA | 201 359 398 |
| *rbcL* | -3234.17 | -3233.13 | 0.353 | 449 456 | NA | 449 | 131 224 226 375 456 |
| *accD* | -3114.01 | -3101.36 | 3.21E-06** | 16 24 28 73 ***81*** ***225*** | NA | ***81*** 136 ***225*** 260 | 17 20 34 64 ***81*** 82 224 ***225*** 229 |
| *psaI* | -249.32 | -249.20 | 0.887 | NA | NA | NA | NA |
| *ycf4* | -1389.98 | -1389.98 | 1 | NA | NA | NA | 67 156 |
| *cemA* | -1551.20 | -1551.20 | 1 | NA | NA | NA | NA |
| *petA* | -2280.11 | -2280.11 | 1 | NA | NA | NA | 229 |
| *psbJ* | -248.41 | -247.68 | 0.482 | 25 | NA | NA | NA |
| *psbL* | -201.12 | -201.12 | 1 | NA | NA | NA |  |
| *psbF* | -207.31 | -206.91 | 0.670 | NA | NA | NA | NA |
| *petG* | -172.05 | -172.05 | 1 | NA | NA | NA | NA |
| *psaJ* | -281.56 | -281.50 | 0.942 | NA | NA | NA | NA |
| *rpl33* | -571.96 | -571.44 | 0.594 | 23 | NA | NA | NA |
| *rps18* | -1125.79 | -1116.73 | 0.000116** | 16 18 91 92 93 94 97 | NA | NA | 16 18 24 54 |
| *rpl20* | -1062.29 | -1057.43 | 0.00775** | ***75*** | NA | 23 ***75*** | 23 48 ***75*** 104 |
| *clpP* | -2245.28 | -2241.19 | 0.0167* | 59 71 ***128*** ***131*** 133 135 165 168 | NA | ***128*** ***131*** 184 | 81 ***128*** 130 ***131*** |
| *psbB* | -3429.16 | -3429.07 | 0.914 | 494 | NA | NA | 31 184 |
| *psbT* | -182.87 | -182.87 | 1 | NA | NA | NA | NA |
| *psbH* | -570.94 | -569.82 | 0.326 | 12 13 | NA | NA | 15 |
| *petB* | -1312.57 | -1311.51 | 0.346 | 112 | NA | NA | 2 |
| *petD* | -1091.87 | -1091.47 | 0.670 | 2 | NA | NA | 2 |
| *rpoA* | -2515.01 | -2515.01 | 1 | NA | NA | NA | 3 150 188 236 241 |
| *rps11* | -1137.87 | -1137.81 | 0.942 | NA | NA | NA | 51 |
| *infA* | -927.19 | -926.96 | 0.794 | NA | NA | NA | 29 73 |
| *rps8* | -1058.46 | -1057.53 | 0.394 | NA | NA | NA | 2 |
| *rpl14* | -908.21 | -908.21 | 1 | NA | NA | NA | 54 |
| *rpl16* | -1095.73 | -1088.79 | 0.000968** | 1 66 74 87 88 121 129 133 | NA | NA | 76 83 117 129 |
| *rps3* | -1812.36 | -1811.65 | 0.491 | NA | NA | NA | 80 107 |
| *rpl22* | -521.16 | -510.43 | 2.187E-05** | 44 | NA | NA | NA |
| *rpl2* | -1525.22 | -1523.77 | 0.234 | 179 219 | NA | NA | 109 249 |
| *rpl23* | -578.92 | -576.59 | 0.0973 | 17 93 | NA | NA | NA |
| *ycf2* | 13974.89 | -13884.04 | 0** | 3 11 41 50 103 110 161 195 ***203*** 204 227 230 233 237 ***238*** 239 ***241*** 242 243 ***249*** 252 254 255 257 ***258*** 265 269 274 ***275*** 276 278 281 287 292 299 301 304 ***306*** 319 321 323 327 328 329 330 332 333 335 336 344 ***346*** ***366*** 391 444 583 587 613 ***645*** 660 663 664 ***666*** 672 681 682 687 689 691 692 695 731 756 768 775 815 819 928 ***959*** 966 967 968 985 986 991 1022 1104 1157 1159 1183 1227 1233 1235 1240 1241 1286 1287 ***1288*** 1293 1294 1315 1336 1359 1372 1376 1378 1379 1383 1386 1392 1400 1403 1404 1405 | NA | ***203*** ***238*** ***241*** ***249*** ***258*** ***275*** ***306*** ***346*** ***366*** 391 625 ***645*** ***666*** ***959*** ***1288*** | 105 161 ***203*** 204 205 206 207 209 228 ***238 241*** 245 ***249*** 251 254 ***258*** 261 265 ***275*** 287 290 292 ***306*** 319 323 327 329 330 336 338 344 ***346*** ***366*** 444 527 589 625 ***645*** 658 664 ***666*** 689 692 702 718 732 775 807 826 832 842 928 ***959*** 968 1220 1221 1223 1224 1225 1227 1229 1232 1233 1234 1235 1237 1241 1279 ***1288*** 1359 1361 1376 1379 1392 1402 |
| *rps7* | -1096.46 | -1090.62 | 0.00291** | 14 19 24 43 67 68 80 81 83 93 | NA | 43 | 43 174 |
| *ccsA* | -2866.01 | -2861.49 | 0.0109* | 38 172 183 191 | NA | 177 226 | 38 191 226 289 306 |
| *psaC* | -538.18 | -538.14 | 0.961 | 70 | NA | NA | 70 |
| *rps15* | -740.51 | -740.05 | 0.631 | NA | NA | NA | 32 63 |
| *ycf1* | -1472.00 | -1468.29 | 0.0244* | 40 89 92 | NA | NA | 6 27 38 105 106 |
| *psbN* | -271.89 | -271.89 | 1 | NA | NA | NA | NA |

Note: *Signiﬁcant at 5% level; ** signiﬁcant at 1% level; Underlined, italicized, and bold represent sites identified by at least three methods
