# Supplementary figures and images for: Comparative Chloroplast Genomes of Zosteraceae Species Provide Adaptive Evolution Insights Into Seagrass
Source: Front Plant Sci. 2021 Sep 23;12:741152. doi: 10.3389/fpls.2021.741152 (PMC8495015; doi:10.3389/fpls.2021.741152)

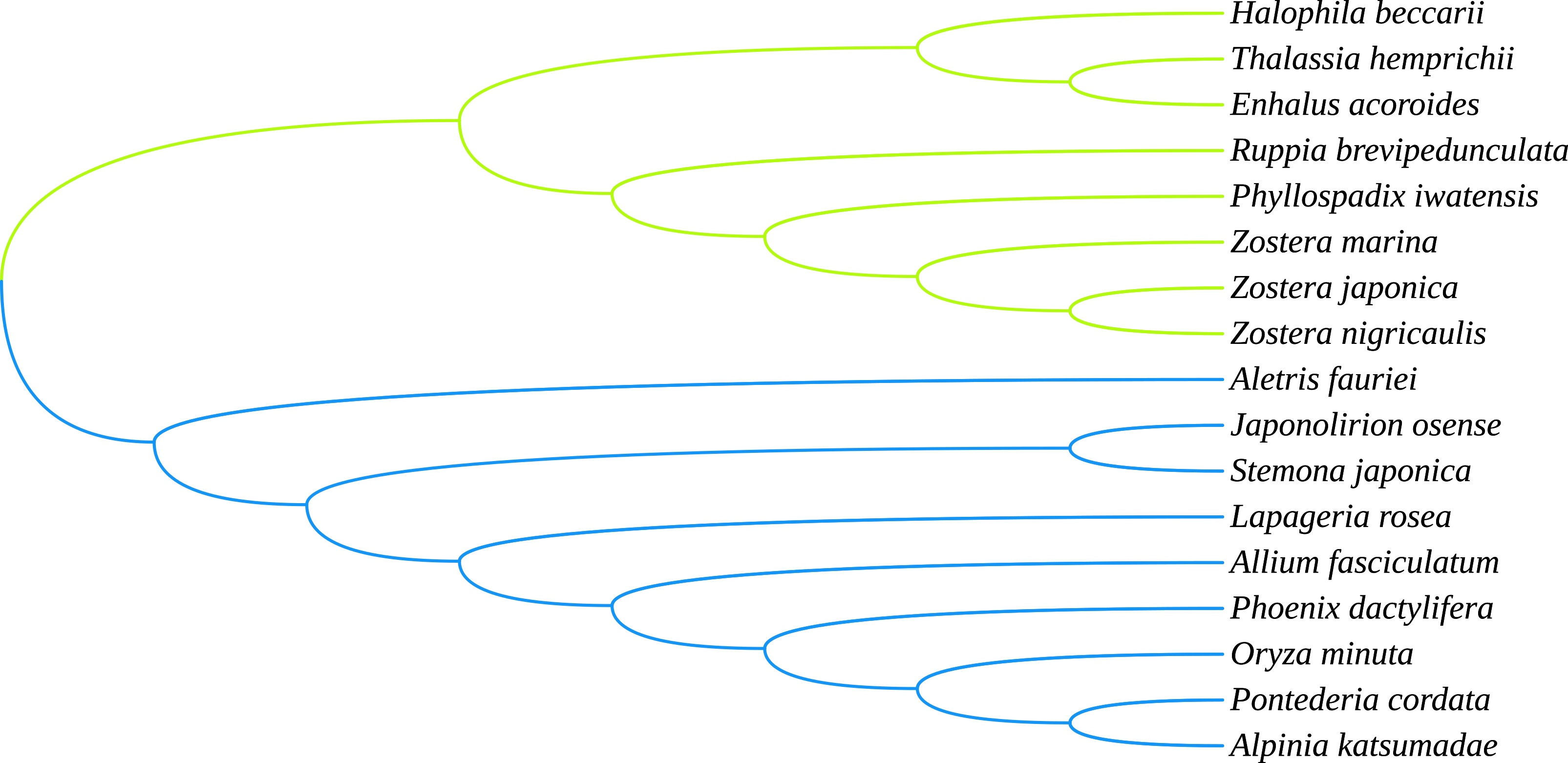

Supplement: Supplementary Figure 1 — Phylogenetic trees of 17 monocotyledonous species. [file Data_Sheet_1.zip › Supplementary Figure S1.JPEG]
